# EEG Spectral Dynamics of Video Commercials: Impact of the Narrative on the Branding Product Preference

**DOI:** 10.1038/srep36487

**Published:** 2016-11-07

**Authors:** Regina W. Y. Wang, Yu-Ching Chang, Shang-Wen Chuang

**Affiliations:** 1 Design Perceptual Awareness Lab (D:PAL), National Taiwan University of Science and Technology (Taiwan Tech), Taipei, Taiwan; 2 The Department of Industrial and Communication Design, National Taiwan University of Science and Technology (Taiwan Tech), Taipei, Taiwan; 3 Taiwan Building Technology Center, National Taiwan University of Science and Technology (Taiwan Tech), Taipei, Taiwan

## Abstract

Neuromarketing has become popular and received a lot of attention. The quality of video commercials and the product information they convey to consumers is a hotly debated topic among advertising agencies and product advertisers. This study explored the impact of advertising narrative and the frequency of branding product exposures on the preference for the commercial and the branding product. We performed electroencephalography (EEG) experiments on 30 subjects while they watched video commercials. The behavioral data indicated that commercials with a structured narrative and containing multiple exposures of the branding products had a positive impact on the preference for the commercial and the branding product. The EEG spectral dynamics showed that the narratives of video commercials resulted in higher theta power of the left frontal, bilateral occipital region, and higher gamma power of the limbic system. The narratives also induced significant cognitive integration-related beta and gamma power of the bilateral temporal regions and the parietal region. It is worth noting that the video commercials with a single exposure of the branding products would be indicators of attention. These new findings suggest that the presence of a narrative structure in video commercials has a critical impact on the preference for branding products.

Whether video commercials can successfully promote products has been a focus of attention in the industry and in academia^1^. In addition, sometimes there is a gap between the design of the video commercial and the needs of the advertiser. In other words, advertising agencies hope to promote their branding products using creativity^2^,^3^, whereas the advertisers are more focused on product performance and information regarding all of the branding products that is conveyed to the public^4^,^5^. This conflict can be resolved by analyzing the views of the consumer or the audience.

We attempted to clarify the significance of the narrative and of the frequency of branding product exposure in video commercials. The experiment utilized the presence of the narrative and the frequency of branding product exposure in the video commercial as independent variables. We considered the audience’s response, as measured by preference for the video commercial and the branding product, and branding product awareness. This study aimed to explore the effects of the “narrative” and the “frequency of branding product exposure” on the audience’s preference for the video commercials and the branding products.

The concept of a “narrative” originated from literary theory^6^,^7^,^8^. After further development by modern marketing and film theory^9^,^10^, a “narrative” is no longer limited to spoken or written accounts of connected events. It is about a “story”^8^, and “a representation of a particular situation or process in such a way as to reflect or conform to an overarching set of aims or values”^11^.

Barthes proposed that the application of narrative structure can be expanded to non-verbal creations^12^. For example, films, comics, advertisements, and design all have a narrative structure, which refers to the structure of the story in chronological order within a period of time^13^. Todorov proposed that the narrative is a process of causal transformation. The basic narrative consists of five phases: equilibrium, disruption, recognition of the disruption, attempts to repair the disruption, and return to the equilibrium^6^,^7^.

We found that video commercials mostly use a set of visuals to form a story to present a branding product^14^. The goal is to obtain the audience’s impression and promote sales. On the other hand, the form of story conveyed by the video commercials is related to the expectations of the product clients and advertising agencies. Whether the consumer understands these visual stories and consequently has a positive attitude toward the branding product is the core issue of our study.

“Advertising narrative” has been studied by researchers in the fields of marketing and psychology. The manners in which the plot and the script structure affect consumers’ gradual understanding of the branding product are discussed. In addition, researchers try to increase the audience’s attention in order to better convince consumers using advertising^15^,^16^,^17^,^18^,^19^. Information regarding the branding products, including their existence, features, and price, is conveyed during advertising. The image of the branding product is expected to be established by repeatedly playing the commercial while the audience’s attention and interest are captured in order to promote sales^20^,^21^,^22^. Multiple or long-term product exposures are considered to have a positive impact on brand recall and on the consumers’ preference for a specific brand^23^,^24^,^25^.

By understanding the consumer’s needs, marketers set communication goals and desired effects^26^,^27^,^28^. Kotler, who is a marketing researcher, summarizes the process as a three-stage “response hierarchy model”: (1) cognitive stage: product awareness is raised in consumers, who extract information to remember and understand the advertising messages; (2) affective stage: positive emotions and preferences are created in the consumers for the advertising content; (3) behavioral stage: consumers, driven by the advertisements, generate the desire to purchase, and act on it^5^. The degree of preference for the advertisement is considered to be the best measure of advertising effectiveness and communication^29^. A popular advertisement can enhance the positive response to advertising information and a brand^30^,^31^. In this study, this measure was used as the indicator for the electroencephalography (EEG) experiment and for the behavioral data investigation.

The use of EEG analysis to explore the effect of advertising has been frequently adopted in recent years^32^,^33^. It is an emerging and popular research method in the fields of advertising and marketing and has given rise to neuromarketing^34^,^35^. EEG experiments can immediately assess the cognitive status of a subject watching video commercials. The intrinsic results help to understand the decision-making behaviors of consumers with high accuracy^36^,^37^,^38^,^39^,^40^.

During the cognitive stage of watching video commercials, the parietal region receives sensory stimuli and messages from different brain regions^5^,^41^. The series of processes involved in cognitive integration includes feature binding, which refers to a nerve stimulator interpreting the representation of the stimulus according to its physical characteristics or personal experience^42^. The gamma rhythms produced in the temporal lobe of the brain are mainly responsible for high-level visual stimulation and are related to high-level cognitive integration^43^. In the cognitive process involved in understanding objects, the increased synchronization of the temporal lobe with the parietal regions, as well as high-frequency beta- and gamma-band responses, indicate the visual response and high-level, multimodal semantic integration^44^.

During the affective stage, the emotions and feelings brought on by the video commercials in the audience affect the preference decision for the commercials^5^,^45^. Past studies have reported that the ventromedial prefrontal cortex is an area important for emotion and plays an important role in translating commercial images into brand preferences^46^. When observing participants watching video commercials, Vecchiato found that asymmetric theta band activities in the left and right frontal lobes are related to the degree of pleasure generated by the video commercials^32^. Lindsen *et al*. also found that enhanced theta band activity in the frontal lobe is associated with positive emotions and subjective preference decisions^47^. In addition, Ambler *et al*. found that the observation of emotional commercials may increase activity in the orbitofrontal region and the amygdala^33^. Damasio believed that the emotional memory processing functions of the amygdala and the insula, which are the decision-making regions, and the limbic region of the frontal lobe, have important correlations with decision-making^48^.

In this study, the views of the consumers or the audience were used to explore controversial issues. The narratives of video commercials and the frequency of branding product exposure were used to explore the EEG dynamics of cognitive integration and the consumer’s preference decision for the advertising stimulus. The stimuli and the experimental design are shown in Fig. 1.

## Results

### Behavioral Results

We studied the two independent variables narrative structure and branding product exposure. We classified the commercials into four types of stimuli based on these variables (Fig. 2). Independent samples t-tests were performed to analyze branding product preferences for the two variables (Fig. 2A). The results indicated that the branding product preference of narratively structured video commercials (mean [M] = 3.21, standard deviation [SD] = 0.86) was higher than that of non-narratively structured video commercials (M = 3.03, SD = 0.91). The difference between these two types of videos was statistically significant (t = −2.747, p = 0.006). Branding product preference for video commercials with multiple exposures (M = 3.19, SD = 0.86) was higher than that for video commercials with a single exposure (M = 3.05, SD = 0.91). The difference between these two groups was also statistically significant (t = 2.115, p = 0.035).

A one-way ANOVA was performed to compare the effects of the four different types of stimuli generated using the two independent variables (Fig. 2B). The branding product preference of the narratively structured multiple exposure (NS-ME) video commercials (M = 3.39, SD = 0.77) was significantly higher than the preference scores for the other three types of advertising stimuli: non-narratively structured single exposure (NNS-SE) (M = 3.12, SD = 0.90, p = 0.037), narratively structured single exposure (NS-SE) (M = 1.90, SD = 0.92, p = 0.000), and non-narratively structured multiple exposure (NNS-ME) video commercials (M = 2.95, SD = 0.91, p = 0.000), among which there were no significant differences.

An independent samples t-test was also performed to assess video commercial preference (Fig. 2C). The results indicated that the video commercial preference for narratively structured video commercials (M = 3.22, SD = 1.01) was higher than that for commercials without a narrative structure (M = 2.91, SD = 0.89). The difference between these two types of commercials was significant (t = −5.122, p = 0.000). The results of the analysis of the effect of the frequency of branding product exposure indicated that there was no significant difference in video commercial preference between video commercials with multiple exposures (M = 3.08, SD = 0.91) and those with a single exposure (M = 3.04, SD = 1.01) (t = 0.636, p = 0.525).

A one-way ANOVA was performed to compare the effects of the four types of stimuli based on the two independent variables (Fig. 2D). The preference scores for the different types of video commercials were as follows: NS-ME (M = 3.46, SD = 0.70) > NNS-SE (M = 3.10, SD = 0.99), NS-SE (M = 2.98, SD = 0.95) > NNS-ME (M = 2.71, SD = 1.03). The highest and the lowest video commercial preference scores were found in the NS-ME and NNS-ME video commercials, respectively. The difference between these two commercial types was statistically significant (p = 0.000).

### EEG Results

Differences in spectral power in various brain regions are summarized in Fig. 3 and are described in the following three sections: (1) EEG results for the cognitive integration-related brain regions, which are the temporal and parietal regions (Brodmann areas [BAs] 13 and 7); (2) EEG results for the preference decision-related brain regions, which are the left frontal region, the bilateral occipital region, and the limbic system region (BAs 9, 31, and 23); (3) EEG results for the perception and attention-related brain regions, which are the frontal midline and bilateral occipital regions (BAs 32 and 31).

### Cognitive integration-related brain regions: temporal and parietal areas

The cognitive integration-related regions for the 2 independent variables were located in the temporal and parietal regions (Fig. 4). The powers of the beta and gamma oscillations in the bilateral temporal (BA 13) and parietal regions (BA 7) generated during the video commercials with narrative structures were significantly higher than those generated during commercials with no narrative structures (p < 0.001). While assessing the influence of branding product exposure on the right temporal region and the parietal region, we found that the powers of the theta oscillations generated during video commercials with multiple exposures were significantly higher than those generated during commercials with a single exposure (p = 0.002 in BA 7; p = 0.036 in BA 13).

Additionally, we observed that the power of the theta oscillations generated during video commercials with a narrative structure was significantly higher than those induced by videos with no narrative structure (p = 0.000). The power of the beta oscillations in the parietal region during video commercials with a single exposure was significantly higher than those generated during videos with multiple exposures (p = 0.04).

### Preference decision-related brain regions: left frontal and bilateral occipital regions and limbic system

Previous studies have indicated that activity in the frontal region, the bilateral occipital region, and the limbic system is related to preference decisions. In this study, the results of the influence of the narrative structure on the left frontal region (BA 9) and the bilateral occipital region (BA 31) indicated that the theta power generated by narratively structured video commercials was significantly higher than that generated by commercials without a narrative structure (p = 0.039 in BA 9; p = 0.002 in BA 31, right occipital; p = 0.018 in BA 31, left occipital) (Fig. 5). The beta band of the bilateral occipital region and the gamma band of the limbic system also had significantly higher frequency responses to video commercials with a narrative structure than those with no narrative structure (p < 0.05). For the impact of branding product exposures, the theta power generated during video commercials with multiple exposures was significantly higher than that generated during video commercials with a single exposure only in the right occipital region (p = 0.000).

### Perception and attention-related brain regions: frontal midline and bilateral occipital regions

We found that the branding products in the video commercials stimulated delta and gamma rhythms in the frontal midline region (BA 32, Fig. 6). The frontal delta and gamma power induced by video commercials with a single exposure were significantly higher than those stimulated by video commercials with multiple exposures (p = 0.013 for delta power; p = 0.004 for alpha power).

Figure 7 shows the significant differences in alpha band activity generated by the four groups of stimuli in the bilateral occipital regions (BA 31) (p < 0.001).

## Discussion

The quality of video commercials and the product information they convey to consumers is a hotly debated topic among advertising agencies and product advertisers. Analyses of behavioral data indicate that the narrative structure of a video commercial has an impact on preference for the branding products and video commercials. In the case of video commercials, the presence of a narrative structure led to the most significant difference. NS-ME commercials were the most favored among the audience and the corresponding products were also the most popular. The least favorite video commercials were NNS-ME commercials. This suggests that the presence of a narrative structure has a critical impact on video commercial preference among the audience.

The EEG results indicate that the presence of a narrative structure generates differences in brain activity in regions responsible for cognitive integration. As can be seen in Fig. 4, in the bilateral temporal and parietal regions, high-frequency beta and gamma powers generated during video commercials with a narrative structure were significantly higher than those generated during non-narratively structured video commercials. This finding is consistent with the proposal by Müller that the power of the gamma band may increase when one receives meaningful stimuli and decreased when the stimuli are meaningless^49^. It is speculated that narrative structures play an important role in the audience’s visual imagery and cognitive integration. The narratively structured video commercials had relatively good feature connecting effects. The subjects’ cognitive integration of the communication and the contents of the video commercials were relatively clear. Lengger *et al*. carried out EEG experiments of art appreciation and concluded that the brain region affected by figurative artwork was different from the region affected by abstract artwork. When the artwork was relatively figurative, or when style information was provided, there were significant activities in the bilateral temporal regions. On the other hand, when the artwork was relatively abstract, or when no style information was provided, the frontal region, especially on the left side, exhibited relatively strong EEG activity^50^. In the present study, the presence/absence of the narrative structure was used as an independent variable in the experiment and was employed to explore differences in the degree of the understanding in the audience. The results of the present study concerning preference for commercials are in agreement with those of Lengger’s study.

Narrative structure influenced cortical activity in decision-making-related regions. In the limbic system, the gamma power produced by narratively structured video commercials was higher than that produced by non-narratively structured video commercials. The limbic system consists of the orbitofrontal cortex, insular cortex, anterior and posterior cingulate cortices, parahippocampal gyrus, hippocampal formation, amygdala, basal forebrain, and hypothalamus^51^. This region is in charge of emotions and memory^52^ and reflects the levels of cognitive activity^53^. It is speculated that video commercials with a narrative structure can produce high levels of emotion and cognitive activity.

Narratively structured video commercials produced significant theta power in the left frontal region. This is consistent with previous findings showing that theta power in the left frontal region is associated with pleasant video commercials and is also related to emotional engagement^32^. It is speculated that consumers have a high level of preference for video commercials with a narrative structure due to the stimulation of a high level of pleasant emotions. Furthermore, the results from the occipital region suggested that the powers of both theta and beta oscillations generated during narratively structured video commercials were higher than those generated during non-narratively structured commercials. This finding is consistent with the results described by Khushaba *et al*., who found the same significant theta and beta band activities in their study on the preference for visual stimulation^54^.

Analysis of behavioral data showed that the frequency of branding product exposure in the video commercial had no significant impact on video commercial preference, but was able to influence the degree of preference for the products. Most likely, the sense of the presence of the branding products enhances the consumers’ preference for them. EEG analysis revealed that the frequency of exposure may produce significant differences in theta power in the cognitive integration-related right temporal and parietal regions, and that the theta power produced by video commercials with multiple exposures was higher than that produced by video commercials with a single exposure. It is speculated that multiple exposures in video commercials can influence cognitive integration. However, the effect sizes of the significant differences are not as large as those associated with narrative structure. Not many preference decision-related brain regions showed significant differences associated with branded product exposure. We only found differences in the right occipital region, but not in other regions. This suggests that our findings may be associated with visual stimulation.

Branding product exposure is associated with significant differences in the frontal delta and gamma rhythms. These findings are related to perception switching and attention^55^. Başar-Eroglu *et al*. found that the enhanced gamma band activity in the frontal region was related to perception switching^56^, and Tiitinen *et al*. found that the 40 Hz gamma band is associated with transient attention^57^. As shown in Fig. 5, the power of the frontal gamma oscillations induced by video commercials with a single exposure were significantly higher than those generated during video commercials with multiple exposures. It is speculated that multiple exposures of the branding product is relatively unlikely to lead to focused attention, while a single exposure is likely to lead to a high level of attention.

In this study, higher alpha power was observed in the bilateral occipital region when the participants watched NNS-ME video commercials (p < 0.01), while lower alpha power was found in the same region while NS-ME video commercials were displayed. Previous studies have shown that occipital alpha rhythms are related to visual perception^58^ and that occipital alpha oscillation is considered to be an indicator of “no message” processing in the visual region^59^. Custódio studied the degree of sensitivity while different ethnic groups watched beer commercials and proposed that occipital alpha rhythms are produced in people who are less interested in the contents of video commercials^60^. Comparisons between video commercial preferences indicated that NS-ME video commercials produce occipital alpha oscillations with the lowest power and the highest degree of preference, while NNS-ME video commercials produce occipital alpha oscillations with the highest power and the lowest degree of preference.

This study found that, (A) The presence of a narrative had a significant positive correlation with preference scores of the branding products and the video commercial. (B) The impact of branding product exposure on the preference for the branding products and the video commercials was in effect conditional; the single factor, frequency of exposure only had a significant positive impact on preference for the branding product and had no significant impact preference for the video commercial. The behavioral data obtained during exposure to the four groups of stimuli produced by combining two variables reveal that preference for the branding products requires an interaction between narrative structure and multiple exposures to branded products. This finding was also confirmed by the EEG results. Narratively structured video commercials led to higher preference decision-related theta power in the left frontal region, theta and beta power in the bilateral occipital region, and gamma power in the limbic system. They also induced significant cognitive integration-related beta and gamma power in the bilateral temporal regions and theta, beta, and gamma power in the parietal region. It is believed that narratively structured video commercials can produce high levels of brain activity related to the preference decision and cognitive integration. The frequency of branding product exposure led to differences in the right occipital, right temporal, and parietal theta bands. However, the levels of significance were not as high as those associated with narrative structure. It is worth noting that the higher frontal delta and gamma power caused by video commercials with a single exposure may be indicators of attention for further investigation.

## Methods

### Participants & Stimuli

Thirty subjects (15 men and 15 women) from Taipei, Taiwan, with an average age of 23.8 years and at least a bachelor’s level education were enrolled in this study. The subjects had a visual acuity of at least 0.8 after vision correction, and had no medical history of color blindness, visual impairment, or any relevant neurological and psychological disorder. The subjects were not addicted to drugs or alcohol and stopped taking stimulants that are able to affect brain activity (e.g., caffeine and alcohol) 48 hours before the experiment. In accordance with the Declaration of Helsinki^61^,^62^,^63^, the study was approved by the Institutional Review Board of Cathay General Hospital. All methods were carried out in accordance with the approved guidelines. Informed consent was obtained from all subjects prior to the experiments.

To incorporate the amendments suggested by the pilot experiment, such as the avoidance of the influence of the subjects’ language comprehension of the story, all of our 30 subjects were from the Taipei metropolitan area, had at least a bachelor’s degree and some professional abilities, and achieved an intermediate proficiency on the National General English Proficiency Test^64^, which is equivalent to 133–173 on the Test of English as a Foreign Language (TOEFL) exam. Due to limitations in financial and manpower resources, we could not measure any other language skills of the subjects. Instead, the subjects’ verbally confirmed that they do not have any language abilities other than those for English and Chinese.

In addition, to ensure that the subjects’ judgments of the experimental stimuli were not affected by their cultural or artistic perception, the stimuli were chosen from the same artistic category and never previously broadcasted in Taiwan^65^. Hence, the stimuli were novel and unfamiliar to the subjects, but were artistically consistent. We selected thirty-two clips from nearly 500 videos from the 2010–2013 Cannes Lions International Festival of Creativity and the London International Advertising Awards. These clips fulfilled our experimental conditions (narrative structure and brand exposure). The average duration of each clip was 50.45 seconds, and included dialogues in English, French, Italian, and Spanish. In a pilot experiment, we confirmed that the products could be identified in all of the clips.

**Figure 1:**
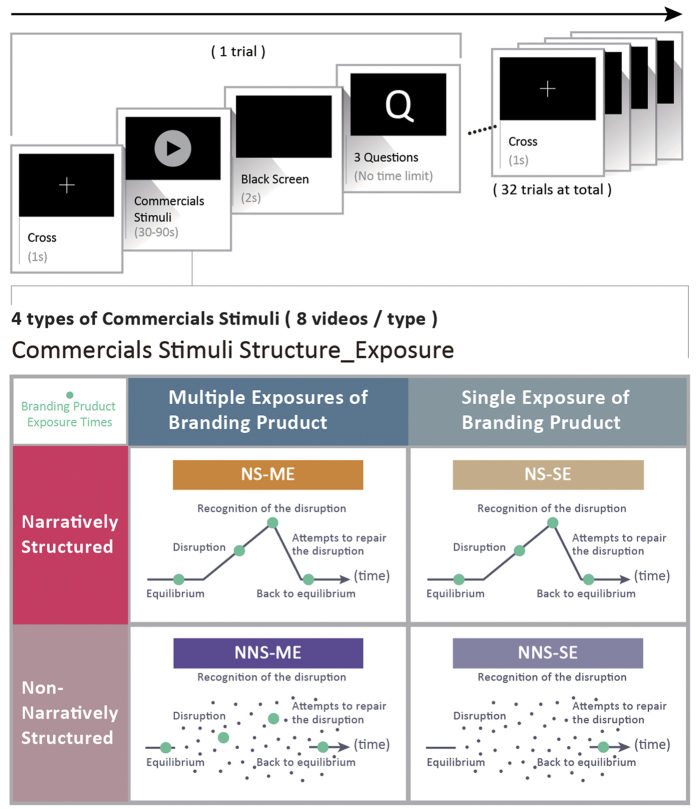
The procedure and the experimental design with the two factors. All video commercials were classified based on narrative structure and branding product exposure: (1) NS-ME–narratively structured with multiple exposures, (2) NS-SE–narratively structured with a single exposure, (3) NNS-ME–non-narratively structured with multiple exposures, (4) NNS-SE–non-narratively structured with a single exposure. Thirty-two video commercials were played randomly with no repeats and there were eight advertising stimuli in each class.

**Figure 2:**
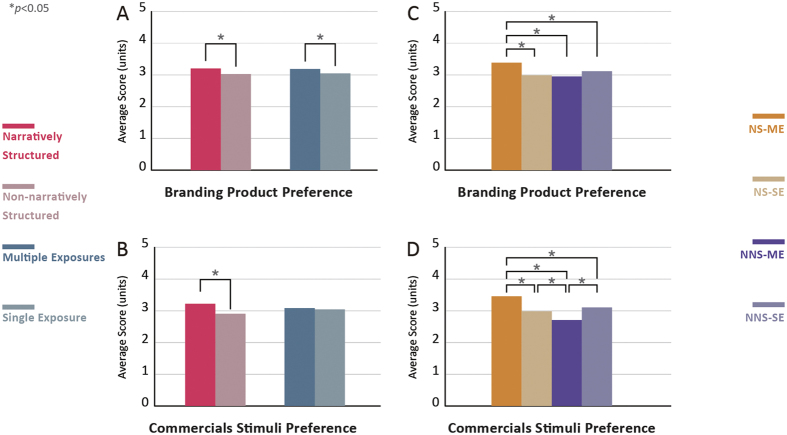
Statistical results of the behavioral data. (**A,B**) The behavioral results of the 2 independent variables as assessed by independent samples t-tests for commercial stimuli and branding product preference. (**C,D**) The behavioral results of the 4 types of commercial stimuli as assessed by a one-way ANOVA for commercial stimuli and branding product preference. The highest preference scores for the branding product and the video commercial were found in the NS-ME group and the difference was statistically significant (p < 0.05). As can be seen in the behavioral results, a greater effect was observed in the narratively structured video commercials.

**Figure 3:**
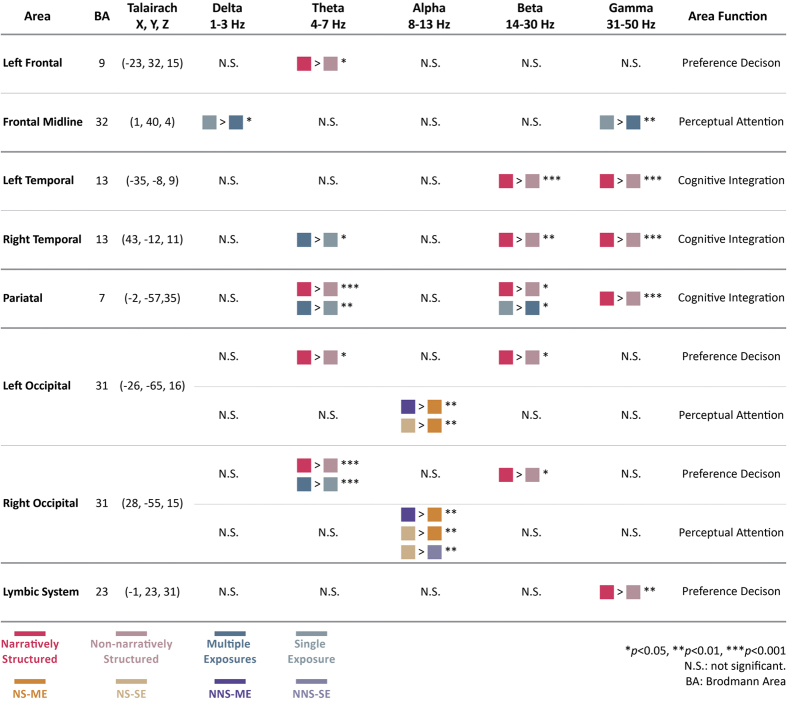
Overview of the EEG results in different brain areas and corresponding brain functions. The EEG results obtained while the subjects viewed the video commercials had significant differences in the spectral power in multiple common regions. These differences were due to the impacts of narrative structure and branding product exposure. The Talairach coordinate of each region centroid were matched to Brodmann areas to assess regional brain activity. A Wilcoxon signed-rank test was carried out to examine whether there were statistically significant differences (p < 0.05).

**Figure 4:**
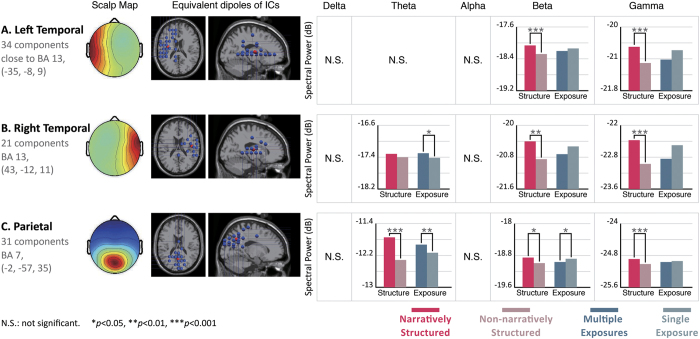
EEG results from 2 cognitive integration-related regions: the bilateral temporal (BA 13) and parietal (BA 7) regions. The frequency bands in each brain region were compared using two factors. The left bar chart represents a single factor, which is the presence vs. absence of a narrative structure. The right bar chart represents a single factor, which is the frequency of branding product exposure. (**A**) Spectral results of the left temporal region, which includes 34 components, showing strong activity in the beta and gamma bands. (**B**) Spectral results of the right temporal region, which includes 21 components. (**C**) Spectral results of the parietal region, which includes 31 components. Both the right temporal region and the parietal region show strong activities in the theta, beta, and gamma bands.

**Figure 5:**
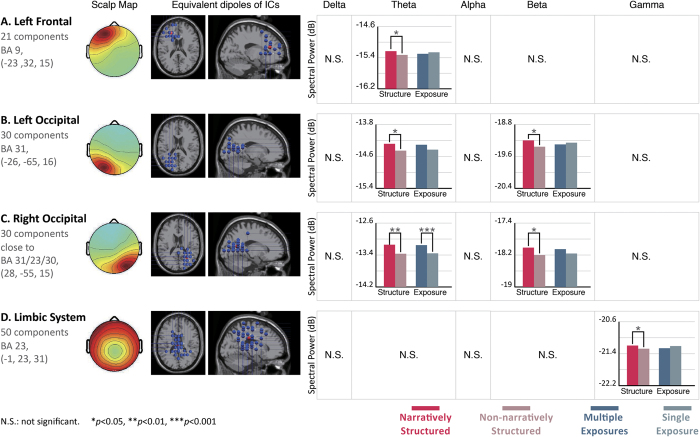
EEG results from 3 preference decision-related regions: the left frontal and bilateral temporal regions, and the limbic system. (**A**) Spectral result of the left frontal region, which includes 21 components, showing strong activity in the theta band. (**B,C**) Spectral results for the left and right occipital regions, both of which include 30 components, and show strong activities in the theta and beta bands. (**D**) Spectral result of the limbic system, which includes 50 components, showing strong activity in the gamma band. The beta band of the bilateral occipital region and the gamma band of the limbic system also showed significantly higher frequency responses to video commercials with a narrative structure than to video commercials with no narrative structure (p < 0.05). For the impact of branding product exposures, the power of the theta oscillations generated during video commercials with multiple exposures were significantly higher than those generated during video commercials with a single exposure. This was only observed in the right occipital region (p < 0.001).

**Figure 6:**
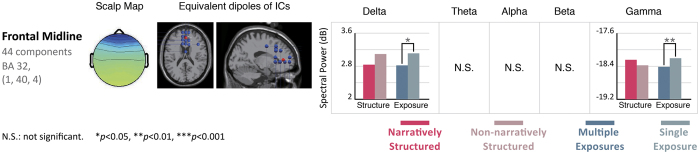
EEG results from the frontal region, which is the perceptual-related region. The frontal region, which includes 44 components, has strong brain activity in the delta and gamma bands associated with differences in the frequency of branding product exposure. There was no effect of narrative structure.

**Figure 7:**
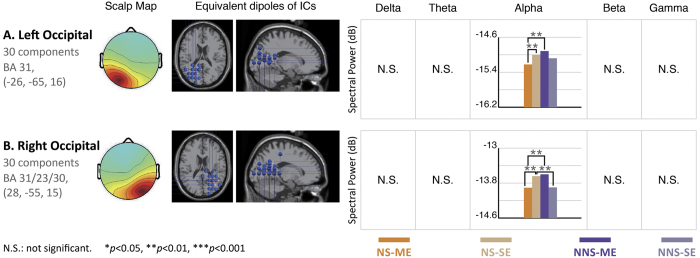
EEG results for the four types of commercial stimuli from the bilateral occipital regions, which are attention-related regions. The bilateral occipital region, which contains 30 components, shows significant differences in brain activities produced by the four types of video commercial stimuli in the alpha band. The highest power in the alpha band was generated during video commercials with no narrative structure and multiple exposures, followed by those obtained while watching a commercial with a narrative structure and a single exposure and those obtained while watching commercials with no narrative structure and a single exposure. The lowest power of the alpha band was presented in the video commercials with a narrative structure and multiple exposures, indicating an apparent correlation between branding product exposures and these two regions (p < 0.01).

The 32 video commercials were classified with narrative structure and branding product exposure as the independent variables in the experiment (Fig. 1). A classifications based on narrative structure was introduced to analyze the plots of the commercials. Those with a full storyline, a plot, and character roles were defined as “having narrative structure”, while those without a full storyline or plots were defined as “having no narrative structure”. The video commercials were also classified based on the frequency of branding product exposure in the video as “multiple exposure” or “single exposure” videos. Multiple exposure videos contained at least two branding product exposures, while single exposure videos contained only one branded product exposure. The duration of each individual branding product exposure was more than 1.5 seconds. Finally, the advertising stimuli were divided into four categories: NS-ME, NS-SE, NNS-ME, and NNS-SE. There were eight advertising stimuli in each class, the average length of each video commercial was 50.45 seconds, and the resolution of the video was 640 × 480 pixels.

### Procedure

This study was performed in the Design Perceptual Awareness Lab (D:PAL) at the National Taiwan University of Science and Technology (Taiwan Tech). During the experiment, external noise, temperature, light, and any other possible disturbances were strictly controlled. The subjects were alone in the experimental room while they watched the videos and answered the questions. During this time, the experimenter was outside the room and observed the state of the subject and the EEG recording. The EEG recording and analysis systems Scan4.3.3 and STIM2 from Neuroscan were used. We used an electrode cap (Quik-cap) and a SynAmps2 amplifier. The 64-channel EEG signal was recorded according to the international 10–10 electrode placement system.

The screen for watching the video commercials was placed on a table with a height of 74 cm. The center of the screen was at a 10–20° viewing angle and was 60–70 cm away from the subject. The instructions were provided before the experiment in order to allow the subjects to relax while they watched the videos and answered questions using a keyboard. Before the start of the video commercials, a cross symbol (+) appeared in the center of the screen for 1,000 milliseconds. Three questions were asked after each video clip: “Do you know what the advertised product is?”, “Do you like this product?”, and “Do you like this commercial?”. Questions two and three were accompanied by four answers representative of the subjects’ preference levels: “high”, “medium”, “low”, and “no feelings”. If the subject was not able to identify the product, the question regarding product preference was skipped. Thirty-two video commercials were played randomly with no repeats and with a break after every eight video commercials. The subject decided on the length of the break before continuing the experiment.

### Behavioral Data Analysis

In the analysis of the behavioral data, the scores for each item were summed up and one-way analysis of variance (ANOVA) was conducted to analyze the scores for branding product preference (“Do you like this product?”) and video commercial preference (“Do you like this commercial?”). The differences between the four groups of advertising stimuli were statistically analyzed. Pairwise comparisons of the data were performed using Scheffe post-hoc tests. Subsequently, the four groups of video commercials were combined into two groups using two independent variables: narrative structure and branding product exposure. Independent sample t-tests were performed to understand the influences of the two independent variables on the preference for the branding product and the video commercials.

### EEG Data Preprocessing

In the process of EEG analysis, a wide range of noises was examined to reduce their impact. This was followed by use of a high-pass filter with a cut-off frequency of 1 Hz and a transition band of 0.2 Hz to remove baseline-drifting artifacts. Subsequently, a low-pass filter with a cut-off frequency of 50 Hz and a transition band of 7 Hz was adopted to remove muscle artifacts and line noise. The sampling rate of the filtered EEG signals was down-sampled to 250 Hz to reduce data storage and analysis time.

### Independent Component Analysis

In order to control for the noise generated during the experiment and to explore the activations of various brain regions during the video commercials, independent component analysis (ICA) was carried out^66^,^67^. The basic assumption of the ICA is that the EEG signals collected from an electrode are a weighted linear mixture of electrical potentials projected instantaneously from distinct, independent brain sources^66^,^67^. First, the EEG signals generated when the subjects answered questions were removed. Then, the activities observed during the observation of the video clips were selected in order to reduce the impact of noise on the EEG signal. Infomax ICA algorithms in EEGLAB^68^ were adopted to separate the 64-channel EEG signal for each subject into independent components. The weight of each individual component electrode was used to obtain the scalp map of the independent components. This represented the regional locations of the independent components, which allowed for a more accurate determination of the source brain area of the activation. The ICA was able to effectively remove the impacts of eye blinking, noise, and muscle activity from the EEG signal^69^,^70^,^71^,^72^,^73^. Finally, the corresponding equivalent dipole location of the independent component in the brain was calculated using DIPFIT2 routines^74^ and was used in further analyses.

### Independent Component Clustering and Spectrum Analysis

To understand common regional brain activation patterns in the 30 subjects, the K-means method^75^ was employed to cluster the scalp maps, power spectral densities, and dipole locations of the independent components^58^,^76^. The independent components of the 30 subjects were clustered into 13 common regions: frontal midline, left frontal, right frontal, central midline, left temporal, right temporal, left motor, right motor, parietal, left occipital, occipital midline, right occipital, and limbic system. Finally, the Talairach coordinate of each region centroid^77^,^78^ was matched to the corresponding Brodmann area (BA)^79^.

Finally, four groups of stimuli were used as variables and fast Fourier transform (FFT)^80^ was used to convert EEG activities to frequency responses. The power spectra of the independent components of each frequency band in the different brain regions were compared and Wilcoxon signed-rank tests were carried out to examine whether there were statistically significant differences (p < 0.05).

## Additional Information

**How to cite this article**: Wang, R. W. Y. *et al*. EEG Spectral Dynamics of Video Commercials: Impact of the Narrative on the Branding Product Preference. *Sci. Rep*. **6**, 36487; doi: 10.1038/srep36487 (2016).

**Publisher’s note**: Springer Nature remains neutral with regard to jurisdictional claims in published maps and institutional affiliations.
